# Anticholinergic drugs and functional, cognitive impairment and behavioral disturbances in patients from a memory clinic with subjective cognitive decline or neurocognitive disorders

**DOI:** 10.1186/s13195-017-0284-4

**Published:** 2017-08-01

**Authors:** Virginie Dauphinot, Christelle Mouchoux, Sébastien Veillard, Floriane Delphin-Combe, Pierre Krolak-Salmon

**Affiliations:** 1 Centre Mémoire de Ressources et de Recherche (CMRR) de Lyon, Hôpital des Charpennes, Hospices Civils de Lyon, 27 rue Gabriel Péri, 69100 Villeurbanne, France; 2 Centre de Recherche Clinique (CRC) – VCF (Vieillissement–Cerveau–Fragilité), Hôpital des Charpennes, Hospices Civils de Lyon, Villeurbanne, France; 3 Hospices Civils de Lyon, Groupement Hospitalier, Service pharmaceutique, Lyon, France; 4 Université Lyon 1, INSERM, U1028; UMR CNRS 5292, Centre de Recherche en Neurosciences de Lyon, Lyon, France

**Keywords:** Cognitive disorders and dementia, Functional disorders, Adverse effects, Neuropharmacology

## Abstract

**Background:**

Drugs with anticholinergic properties may be associated with various adverse clinical effects. The relationship between the anticholinergic (AC) burden and functional, global cognitive performance and behavior disturbances was assessed among elderly patients.

**Methods:**

A cross-sectional study was conducted between January 2012 and June 2014 in a memory clinic among outpatients living at home and with subjective cognitive decline (SCD) or neurocognitive disorders (NCD). The AC burden was measured using the Anticholinergic Drug Scale (ADS), the Anticholinergic Risk Scale (ARS), the Anticholinergic Cognitive Burden (ACB), Chew’s score, Han’s score, and the number of drugs with AC activity. Functional, cognitive performance and behavior disturbances were assessed using the Instrumental Activities of Daily Living (IADL) scale (IADL), the Mini Mental State Examination (MMSE), and the Neuropsychiatric Inventory (NPI).

**Results:**

Among 473 included patients, 46.3% were at major NCD. Patients took on average 5.3 ± 2.6 drugs. MMSE was lower when Han’s score (*p* = 0.04) and number of AC drugs were higher (*p* < 0.001). IADL was lower when AC burden was higher, whatever the AC measurement. NPI was higher when ACB, Han’s score, and number of AC drugs were higher. After adjustment, all AC scores remained associated with IADL, while Han’s score and number of drugs with AC remained associated with the MMSE.

**Conclusions:**

In patients with SCD or NCD, AC burden is associated with lower functional score, whereas the cross-sectional association between AC burden and cognitive performance or behavioral disturbance varies according to AC scores. Particular attention should be paid when prescribing drugs with AC properties, especially among patients with memory complaints.

## Background

Drugs with anticholinergic (AC) properties are taken for a broad range of conditions, such as antihistamines for allergy, anticholinergics for urinary incontinence, prostatic hypertrophy, or obstructive lung disease, antipsychotics for psychosis, antidepressants for depression, and benzodiazepines for sleep disorders or anxiety or behavioral symptoms in dementia [1]. While some drugs are well known to have these AC properties (e.g., amitriptyline, doxepin, oxybutynin), numerous drugs have an unexpected AC activity that is not targeted for clinical effect (e.g., digoxin, furosemide). Drugs with AC activity may be associated with various adverse clinical effects including falls, delirium, cognitive and physical function impairment, and all-cause mortality [2–8]. Longitudinal studies have also shown that exposure to AC may increase the risk for developing mild cognitive impairment (MCI) and dementia [9–15]. Most of the studies have been conducted among elderly people in primary prevention, whereas studies assessing relationships between AC and health outcomes in ambulatory patients with subjective cognitive decline (SCD), mild or major neurocognitive disorders (NCD), also known as MCI or dementia, including Alzheimer’s disease or related diseases (ADRD) remain scarce [16, 17]. Although the drug management of behavioral disorders associated with NCD includes the prescription of psychotropic drugs, this prescription may increase the adverse events associated with AC exposure among these patients which makes it particularly challenging for physicians [18]. Furthermore, the adverse effects of AC drugs may be higher among patients with NCD, especially the central anticholinergic adverse effects. These increased effects might be due to physiological changes, modifications in drug metabolism, pharmacodynamics, and pharmacokinetics that are observed among aging patients with cognitive impairment.

Several scales or scores have been developed to evaluate the anticholinergic burden. These scales take into account the serum anticholinergic activity, in vitro measurement of the muscarinic receptor affinity, clinical side effects, expert opinion, and global review of the literature. However, manifold differences exist in scale development, in the selection of AC drug, and in the evaluation of anticholinergic burden. Different proportions of anticholinergic prescriptions in elderly patients with dementia have been highlighted, i.e., between 17% and 50% depending on the anticholinergic prescription measure and the population characteristics [16, 19–22]. Previous studies comparing different AC scores and their associations with health outcomes have also highlighted discrepancies in the results [23]. Moreover, this comparison has not yet been conducted among patients from a memory clinic at different stages of cognitive disorders [2, 3, 24, 25].

We have hypothesized that patients with higher AC burden had lower cognitive and functional performance, and higher behavioral disturbances, with these three outcomes being analyzed separately.

## Methods

### Aim of the study

This study aimed at assessing the cross-sectional relationship between the anticholinergic burden, evaluated with five AC scores, and the number of drugs with AC properties, and three symptoms related to cognitive disorders: functional and global cognitive performance, as well as behavior disturbances, among elderly patients.

### Study design, setting, and population

A cross-sectional study was conducted on a cohort of 473 outpatients visiting a memory clinic of the Clinical and Research Memory Centre of Lyon (Charpennes Hospital, University Hospital of Lyon, France), between 1 January 2012 and the 30 June 2014. The study population included a sample of consecutive patients who expressed a cognitive complaint, either themselves or by one of their relatives. The inclusion criterion in this study was patients with at least one drug prescribed either by the general practitioner or the neurologist/geriatrician.

### Data collection

Sociodemographic and clinical data were reported in an electronic case report form (eCRF), by the secretaries or the nurses of the memory clinic, who has been trained to input the data in the forms.

### Medication and anticholinergic exposure assessment

Medications taken by the patient at the time of the visit to the memory clinic were collected using the prescription of the referent general practitioner and the prescription of the neurologist or geriatrician at the memory clinic. In particular, the generic or brand name of the drug, its dose and frequency and the active substances were noted. AC burden was measured using five scales, previously developed using various methodologies to determine the anticholinergic burden [26], as well as the number of drugs with AC properties. For each patient, the Anticholinergic Drug Scale (ADS), the Anticholinergic Risk Scale (ARS), the Anticholinergic Cognitive Burden (ACB), and Chew’s score and Han’s score were calculated [1, 27–31]. Each drug for each patient was weighted according to the specific scale and a total score for each patient was calculated by summation.

The ADS was developed based on the level of serum anticholinergic activity (SAA) of 102 drugs. The ADS has been validated using a group of aged institutionalized patients [27]. Drugs were scored from 0 to 3 as follows: 0, no known anticholinergic property; 1, anticholinergic potential demonstrated by in vitro binding studies with muscarinic receptors; 2, anticholinergic effect sometimes noted, usually at high doses; 3, high potential anticholinergic effect demonstrated. The ARS is based on a literature search and expert review of the 500 most prescribed medications within the Veterans Affairs Boston Healthcare System [28]. The calculation of dissociation constants for muscarinic acetylcholine of the drugs have been taken into account. The ARS excludes topical, ophthalmic, otology, and inhaled medication preparations. Medications were scored from 0 to 3 as follows: 0, no anticholinergic property known; 1, anticholinergic potential, low risk; 2, anticholinergic average, moderate risk; 3, high anticholinergic potential, major risk.

The ACB was published in the frame of the Aging Brain Program of the IU Center for Aging Research [29]. It is designed as a tool for practitioners to identify the severity of adverse effects of anticholinergic medication, particularly on cognition. It was developed from a broad review of the medical literature (Medline) on the level of serum anticholinergic activity and anticholinergic scale evaluated by a clinician using radioimmunoassay affinity between drugs and muscarinic receptors in rats. Thereby, ACB includes a list of 88 drugs with AC properties. Drugs are scored from 0 to 3 as follows: 0, no anticholinergic property known; 1, anticholinergic drugs with a potential effect on cognition, demonstrated in vitro by its affinity for the muscarinic receptor or by calculating the level of SAA, but without evidence of clinically relevant adverse cognitive effects; 2, moderate or severe anticholinergic effect on cognition which is clinically clear but not causing confusion; 3, moderate or severe anticholinergic effect on cognition which is clearly established clinically and causing confusion. Scores 2 and 3 are differentiated by the ability to cause delirium and pass through the blood-brain barrier.

The Chew’s list is based on in vitro SAA measurements of drugs with effective or minimal anticholinergic activity [1]. Chew’s list scored drugs from 0 to +++ (comprising levels of 0, 0/+ (for drugs with minimal AC activity or with no AC activity at doses across the therapeutic rage but for patients with above average Cmax or receiving supratherapeutic doses that may show some AC activity), +, ++, and +++). In this study, this scale was changed into a numerical score from 0 to 4, where +++ corresponds to 4, ++ to 3, + to 2, and 0/+ to 1.

The Han’s score was developed to assess potential effects of anticholinergic drug use on the severity of delirium in the elderly patients, with scores ranging from 0 (no anticholinergic effect known) to 3 (strong effect which is clearly established clinically) [30]. The Han’s score is based on geriatrician expert opinion, with combined information about the effects of anticholinergic drugs available in the literature and their own rating for the drugs without previous anticholinergic scoring in order to determine the anticholinergic loads.

The five AC scores were studied as discrete binary variables to determine proportions of patients exposed to AC burden and as interval variables to account for the cumulative exposure to AC burden.

### Clinical outcomes

Functional and global cognitive performances, as well as the behavioral and psychological symptoms of dementia (BPSD), were evaluated by a neurologist, a geriatrician, a neuropsychologist, or a trained nurse during the patient’s visit to the memory clinic, according to standardized procedures. The functional performance was assessed using the Instrumental Activities of Daily Living (IADL) scale during the interview with the primary caregiver combined with the memory visit. The IADL assessed eight instrumental activities, the total score ranging from 0 (dependent) to 8 (independent) [32]. Global cognitive performance was assessed with the Mini Mental State Examination (MMSE), ranging from 0 to 30 [33]. The BPSD were assessed using the Neuropsychiatric Inventory (NPI) during the memory visit [34]. A higher overall NPI score (maximum 144) indicates more severe behavioral disorders.

### Other characteristics

The diagnostic stage and the etiology were determined by the neurologist or the geriatrician at each visit. Patients with cognitive complaint and absence of objective evidence are considered as patients with subjective cognitive decline (SCD) [35]. Mild and major neurocognitive disorders (NCD) are identified using the Diagnosis and Statistical Manual of mental disorders (DSM-V) nomenclature [36]. The McKhann et al. [37] and the Albert et al. [38] criteria are used to establish the diagnostic stage of mild or major NCD in AD. The etiologies are identified as follows: AD, AD with cerebrovascular component, vascular dementia (NINDS-AIREN criteria), Lewy body disease, frontotemporal dementia, other pathologies leading to a progressive cognitive impairment (including chronic hydrocephalus, progressive supranuclear palsy, corticobasal degeneration, and unclassified dementia), Parkinsons’s disease, psychiatric disorders (including psychoses, anxious disorder, isolated depression disorder, recurrent depressive disorder, bipolar disorder, and unclassified psychiatric disorders), and others disorders (including other neurological diseases such as tumor and aneurysm, head injury, and organic brain disorder related to the pathology such as metabolic deficiency) [39–41]. The comorbidities of hypertension, diabetes mellitus, and hypercholesterolemia when known at inclusion were collected. Sociodemographic characteristics collected included gender, date of birth, marital status, educational level (middle school without certificate in general education, middle school with certificate in general education, high school, or university/college), and current living situation.

### Statistical analysis

The study population was described using means ± standard deviation (SD) or proportions as appropriate. The exposure to AC burden according to the different scores was described using mean ± SD and proportions of patients with AC burden. The proportion of the most frequent drugs with AC effect was presented as text. The relationship between each outcome, considered as the dependent variables (i.e., MMSE, IADL, NPI) and each AC score as well as the number of drugs with AC properties was assessed using a separate ﻿linear regression model. Hypotheses of application of linear regression models were verified by analyzing the distribution of the residuals of the models which showed skewness and Kurtosis statistics close to zero (normal distribution). The homoscedasticity of the variances was verified using the Levene test. Results were summarized by crude regression coefficients, their 95% confidence intervals, standardized regression coefficients, *p* value, and the coefficient of determination of the models in % (*R* ^2^). Multiple linear regressions were then performed, with an automatic backward stepwise procedure to adjust the estimates for the potential confounding factors of age, gender, educational level, current lifestyle, diagnostic stage, hypertension, diabetes mellitus, hypercholesterolemia, and the total number of drugs, and to eliminate the variables that did not contribute significantly in the models.

All tests were two-tailed. No missing data imputation was performed. A *p* value less than 0.05 was considered statistically significant. All analyses were performed with SPSS statistical software (Statistical Package for the Social Sciences) version 19.0 for Windows (SPSS Inc., Chicago, Illinois, USA).

## Results

A total of 473 patients (mean age 80.6 ± 7 years) were included in the study (Table 1). The population study included patients at major NCD stage (46.3%), mild NCD stage (27.7%), and patients with SCD (26%). For 33.8% of the patients, there was a probable diagnostic of Alzheimer’s disease. Patients took on average 5.3 ± 2.6 different drugs, and on average 1.9 ± 1.7 drugs with AC properties. Patients with major NCD took on average more drugs with AC properties (2.1 ± 1.8) than patients with mild NCD (1.7 ± 1.7) or with SCD (1.6 ± 1.7) (*p* = 0.03). There was no significant difference in the average number of drugs with AC properties between patients with mild NCD and SCD (*p* = 0.75).

**Table 1:** Patient characteristics

Variables	*n*	Mean ± SD or %
Age (years)	473	80.58 ± 7.48
Gender		
Female	291	61.52%
Male	182	38.48%
Educational level		
Middle school without certificate	73	15.43%
Middle school with certificate	174	36.79%
High school	150	31.71%
University/college	76	16.07%
Current lifestyle		
At home with husband/spouse	273	57.72%
At home with relatives	29	6.13%
At home, alone, with relatives in the neighborhood	119	25.16%
At home, alone, without relatives in the neighborhood	30	6.34%
Other lifestyle	22	4.65%
Number of drugs	473	5.27 ± 2.57
Diagnostic stage		
Subjective cognitive impairment	123	26.00%
Mild neurocognitive disorder	131	27.70%
Major neurocognitive disorder	219	46.30%
Diagnostic etiology		
Alzheimer’s disease	160	33.83%
Alzheimer’s disease with cardiovascular component	30	6.34%
Vascular dementia	29	6.13%
Lewy body disease	8	1.69%
Fronto temporal dementia	5	1.06%
Other dementia	8	1.69%
Parkinson’s disease	8	1.69%
Psychiatric disorders	37	7.82%
Others disorders	17	3.59%
Diagnostic not yet established	171	36.15%
Comorbidities		
Hypertension	192	40.60%
Diabetes mellitus	53	11.21%
Hypercholesterolemia	79	16.70%
MMSE		
>20 (mild)	241	50.95%
10–20 (moderate)	181	38.27%
<10 (severe)	26	5.50%
Characteristics considered as continuous scores		
MMSE	448	20.02 ± 6.29
IADL	469	3.60 ± 2.45
NPI	410	18.27 ± 13.40

*MMSE* Mini Mental State Examination, *IADL* Instrumental Activities of Daily Living, *NPI* Neuropsychiatric Inventory

The most frequent drugs with an AC effect prescribed in this study population were: “furosemide”/diuretic (10.1%), “alprazolam”/anxiolytic (9%), “paroxetine”/antidepressant (6.6%), “venlafaxine”/antidepressant (5.9%), “atenolol”/beta-blocking (3.8%), “risperidone”/antipsychotic (2.5%), “digoxin”/cardiac glycoside (2.3%), “olanzapine”/antipsychotic (1.9%), “solifenacin”/drug for urinary incontinence (1.7%), “tramadol”/analgesic opioid (1.5%), “warfarin”/antithrombotic (1.3%), and “amitriptyline”/antidepressant (1.1%). Among the 473 patients, 12.5% had at least one drug with a high AC effect. This proportion was not significantly different according to diagnostic stage: 12.8% in patients with major NCD, 11.5% in patients with mild NCD, and 13% in patients with SCD (*p* = 0.92). In detail, the drugs considered to have a higher AC effect according to the five scores in the study population were “amitriptyline”/antidepressant, “hydroxyzine”/anxiolytic, “olanzapine”/anxiolytic, “paxoretine”/antidepressant, “clozapine”/anxiolytic, “levomepromazine”/anxiolytic, “trihexyphenidyle”/anti-parkinson drug, “oxybutynin”/drug for urinary incontinence, and “clomipramine”/antidepressant.

The proportion of patients with AC exposure, whatever the strength of the AC effect, ranged from 17.1% (ARS) to 49.7% (Chew score) according to the different scores (Table 2). When the scores were considered altogether, 68.7% (*n* = 325) of patients had at least one AC drug. The proportion of AC exposure (42.5%), measured with Han’s score, was significantly higher for patients with major NCD compared to patients at other stages (*p* = 0.01), whereas the proportion of AC exposure was not different between patients with mild NCD and patients with SCD (*p* = 0.93).

**Table 2:** Description of the anticholinergic exposure according to the different scores, and according to the diagnostic stage

		Exposure to anticholinergic, frequency (%)				
	Mean ± SD	Total (*n* = 473)	Major NCD (*n* = 219)	Mild NCD (*n* = 131)	SCD (*n* = 123)	*p* value for difference between diagnostic stage*
ACB	0.78 ± 1.24	188 (39.75%)	98 (44.75%)	47 (35.88%)	43 (34.96%)	0.12
ADS	0.67 ± 0.93	208 (43.97%)	108 (49.32%)	56 (42.75%)	44 (35.77%)	0.05
ARS	0.26 ± 0.67	81 (17.12%)	41 (18.72%)	22 (16.79%)	18 (14.63%)	0.63
Chew’s score	1.09 ± 1.39	235 (49.68%)	118 (53.88%)	58 (44.27%)	59 (25.11%)	0.20
Han’s score	0.57 ± 0.89	168 (35.52%)	93 (42.47%)	39 (29.77%)	36 (29.27%)	0.01^†^

*Unadjusted association

^†^Association between Han’s score and diagnostic stage remained significant (*p* = 0.03) after adjustment for age (*p* = 0.12), gender (*p* = 0.92), total number of drugs (*p* ≤ 0.001), and Neuropsychiatric Inventory (NPI) (*p* = 0.06). Pairwise test showed significant difference between major NCD and mild NCD, and SCD (*p* = 0.01), and no difference between mild NCD and SCD (*p* = 0.93)

*ACB* Anticholinergic Cognitive Burden, ADS Anticholinergic Drug Scale, ARS Anticholinergic Risk Scale, *NCD* neurocognitive disorder, *SCD* subjective cognitive decline

In unadjusted regression analysis, lower MMSE was associated with higher AC exposure measured with Han’s score (regression coefficient *r* = –0.7, *p* = 0.04, standardized regression coefficient = –0.10) and the number of drugs with AC properties (*r* = –0.5, *p* = 0.003, standardized regression coefficient = –0.14) (Table 3). The same association was observed between MMSE and ACB, ADS, ARS, and Chew’s score, but the associations were not significant.

**Table 3:** Unadjusted regression coefficients of MMSE, IADL, and NPI for each anticholinergic scale and total number of drugs (one model for each covariable)

	Outcome 1: MMSE				Outcome 2: IADL				Outcome 3: NPI			
Covariable	*r* (95% CI)	Standardized *r*	*p* value	*R* ^2^	*r* (95% CI)	Standardized *r*	*p* value	*R* ^2^	*r* (95% CI)	Standardized *r*	*p* value	*R* ^2^
ACB	–0.22 (–0.69 to 0.25)	–0.04	0.36	0.2%	–0.27 (–0.45 to –0.09)	–0.14	0.003	1.6%	1.13 (0.04 to 2.21)	0.10	0.04	1%
ADS	–0.37 (–1.00 to 0.26)	–0.06	0.25	0.3%	–0.32 (–0.56 to –0.08)	–0.12	0.009	1.3%	1.08 (–0.36 to 2.53)	0.07	0.14	0.5%
ARS	–0.08 (–0.97 to 0.82)	–0.01	0.86	<0.1%	–0.37 (–0.71 to –0.04)	–0.10	0.03	0.8%	0.52 (–1.50 to 2.53)	0.03	0.62	0.1%
Chew’s score	–0.05 (–0.47 to 0.37)	–0.01	0.81	<0.1%	–0.30 (–0.46 to –0.14)	–0.17	<0.001	2.5%	0.57 (–0.37 to 1.51)	0.06	0.23	0.3%
Han’s score	–0.68 (–1.34 to –0.02)	–0.10	0.04	0.7%	–0.37 (–0.62 to –0.12)	–0.13	0.004	1.6%	1.85 (0.40 to 3.30)	0.12	0.01	1.5%
Number of drugs with AC properties	–0.51 (–0.85 to –0.18)	–0.14	0.003	2%	–0.35 (–0.48 to –0.23)	–0.25	<0.001	6.1%	1.17 (0.43 to 1.91)	0.15	0.002	2.3%
Total number of drugs	0.02 (–0.20 to 0.25)	0.01	0.83	<0.1%	–0.11 (–0.19 to –0.02)	–0.11	0.02	1.2%	0.81 (0.31 to 1.32)	0.16	0.002	2.4%

*AC* anticholinergic, *ACB* Anticholinergic Cognitive Burden, *ADS* Anticholinergic Drug Scale, *ARS* Anticholinergic Risk Scale, *CI* confidence interval, *IADL* Instrumental Activities of Daily Living, *MMSE* Mini Mental State Examination, *NPI* Neuropsychiatric Inventory, r Regression coefficient, R² Coefficient of determination

Lower IADL was associated with higher AC exposure (*r* between –0.4 and –0.3 depending on the AC score). The coefficients of determination were higher for the number of drugs with AC properties and Chew’s score. The total number of drugs was also negatively associated with the IADL (*r* = –0.1, *p* = 0.02).

Higher NPI was associated with higher AC exposure, measured with the number of drugs with AC properties, the Han’s score, or ACB (*r* = 1.2, *p* = 0.002; *r* = 1.9, *p* = 0.01; *r* = 1.1, *p* = 0.04, respectively). The coefficient of determination was higher for the number of drugs with AC properties and Han’s score than the ADS score. The total number of drugs was also positively associated with the NPI (*r* = 0.8, *p* = 0.002). The same associations were observed with the others AC scores without reaching statistical significance.

When the linear regression model was adjusted for age, educational level, and hypertension, Han’s score remained significantly associated with the MMSE (Table 4). After adjustment for age, educational level, total number of drugs, and hypertension, the number of drugs with AC properties also remained associated with the MMSE: for each +1 SD number of drugs with AC, the MMSE was lowered by 0.2 SD, assuming the others variables in the model were held constant. After adjustment for age, gender, educational level, current lifestyle, diagnostic stage, and total number of drugs, all the AC scores and the total number of drugs with AC properties remained negatively associated with the IADL. After adjustment for age, diagnostic stage, and total number of drugs, ACB, Han’s score, and the number of drugs with AC properties were not associated anymore with the NPI.

**Table 4:** Adjusted regression coefficients of MMSE, IADL, and NPI for each anticholinergic score

	Outcome 1: MMSE				Outcome 2: IADL				Outcome 3: NPI			
	*r* (95% CI)	Standardized *r*	*p* value	*R* ^*2*^	*r* (95% CI)^c^	Standardized *r*	*p* value	*R* ^2^	*r* (95% CI)^d^	Standardized *r*	*p* value	*R* ^2^
ACB					–0.23 (–0.40 to –0.07)	–0.12	0.005	28%	0.42 (–0.71 to 1.55)	0.04	0.47	5%
ADS					–0.37 (–0.57 to –0.16)	–0.12	0.005	28%				
ARS					–0.39 (–0.68 to –0.10)	–0.11	0.009	27%				
Chew’s score					–0.25 (–0.39 to –0.11)	–0.13	0.003	28%				
Han’s score	–0.74 (–1.36 to –0.12)^a^	–0.09	0.02	14%	–0.25 (–0.47 to –0.03)	–0.09	0.03	27%	1.03 (–0.47 to 2.52)	0.07	0.18	5%
Number of drugs with AC properties	–0.67 (–1.03 to –0.32)^b^	–0.18	<0.001	16%	–0.27 (–0.39 to –0.16)	–0.20	<0.001	30%	0.67 (–0.12 to 1.46)	0.09	0.10	6%

^a^Adjustment for age, educational level, and hypertension. Gender, current lifestyle, diabetes mellitus, and hypercholesterolemia and total number of drugs were not significant

^b^Adjustment for age, educational level, total number of drugs, and hypertension. Gender, current lifestyle, diabetes mellitus, and hypercholesterolemia were not significant

^c^Adjustment for age, gender, educational level, current lifestyle, total number of drugs, and diagnostic stage. Hypertension, diabetes mellitus, and hypercholesterolemia were not significant

^d^Adjustment for age, diagnostic stage, and total number of drugs. Gender, educational level, current lifestyle, hypertension, diabetes mellitus, and hypercholesterolemia were not significant

*AC* anticholinergic, *ACB* Anticholinergic Cognitive Burden, ADS Anticholinergic Drug Scale, ARS Anticholinergic Risk Scale, *CI* confidence interval, *IADL* Instrumental Activities of Daily Living, *MMSE* Mini Mental State Examination, *NPI* Neuropsychiatric Inventory

## Discussion

In this study among patients visiting a memory clinic with SCD, mild NCD, or major NCD, AC exposure (measured with ADS, ARS, ACB, Chew’s score, Han’s score, and the total number of drugs with AC properties) is independently associated with functional impairment while controlling for age, gender, educational level, current lifestyle, diagnostic stage, and the total number of drugs. For the MMSE and the NPI, the results vary depending on the measure of AC exposure, but the associations show similar trends. Thus, the AC exposure measured with Han’s score and the number of drugs with AC properties is associated with lower cognitive performance, whereas statistical significance is not reached with the others AC scores. AC exposure measured with the ACB, the Han’s scores, and the number of drugs with AC exposure is positively associated with the BPSD assessed with the NPI in the unadjusted analyses, but when the total number of drugs is included in the model the relationships with the AC exposure are no more significant. In this population study, the Han’s score and the number of drugs with AC properties appear to be more related with concomitant impairment in functional and cognitive performance. The fact that the Han’s score is based on expert opinion, and is therefore more clinically focused than ADS and Chew’s scores, may be a possible explanation why it is more associated with the outcomes. However, ARS and ACB scores are also based on expert opinion. As the AC measurement scales depend on the context in which they were developed and proposed (i.e., methodology of assessment, setting, population characteristics, year), these variations may explain the discrepancies of results observed between the AC scores.

In terms of population study, the relationship between AC burden and health disorders was assessed among patients with cognitive complaints at different diagnostic stages whereas, in previous studies, comparison of AC scales has been conducted in elderly people without SCD or ADRD [2, 3, 23]. The inclusion of patients at different stages of cognitive impairment allows us to study whether the effect of AC exposure may differ between these stages. This study shows that the association between AC exposure and the symptoms related to NCD was independent of the diagnostic stage, while the proportion of AC exposure is higher among patients with major NCD as expected. In terms of health outcomes, no previous study has been carried out to study the effect of AC exposure on three different outcomes relevant to ADRD.

In previous studies conducted among elderly people without memory complaints or dementia, and using different methods to measure AC exposure, some authors have made similar observations. AC exposure was associated with increased risk of adverse events or deteriorating health conditions, i.e., falls, hospitalization, all-cause mortality, or worse cognitive and functional performance [2–4]. In contrast, Swami et al. have shown that AC exposure was associated with higher global cognition among a large community-dwelling population of people aged 60 years or more [23]. The use of different scales to estimate the AC exposure leads to a large range of estimated proportion of patients with AC burden. Indeed, in the present study, various proportions of patients exposed to AC were observed depending on the scales. Although this difference is explained by the way the AC scales were used, it highlights the lack of consistency and standards to measure such exposure. Therefore, it appears difficult to compare the proportions of patients exposed to AC burden between different study populations in the absence of a referent AC scale, or because of the use of different thresholds to present this exposure. This difficulty is even greater than the characteristics of the study population that are associated with AC prescription, such as health condition, age, geographical area, and year. However, the proportion of patients with AC burden appears to be higher with increasing severity of the diagnostic stage, with a difference observed between patients with major NCD and the others groups: mild NCD and SCD. In a previous study conducted in France on community-dwelling elderly people without cognitive disorders at inclusion, the proportion of patients with AC burden was estimated at 7.5% [11]. In a study conducted in the USA among elderly demented patients in 2005–2009, the proportion of AC exposure was estimated at around 23% using the ADS scale (level higher than 2) [42]. In another study, the proportion of AC drug use was estimated at 33% among older patients with probable dementia [21]. A higher proportion of AC drug use among patients with major NCD may be explained by the management of BPSD with antipsychotic drugs and with urinary incontinence treated with AC drugs, both these health disorders becoming more frequent with dementia [43, 44].

In another study, Shah et al. found that overall cognitive functions decreased faster among participants who had initiation of medication AC, measured with ACB, compared to those who were never exposed in a population of 896 community-dwelling elderly people without dementia at baseline (mean follow-up of 10 years) [15]. To the contrary, Fox et al. showed that the impact of anticholinergic exposure, also measured with ACB, was not found to be associated with cognitive performance evolution at 18 months follow-up among 224 patients with Alzheimer’s disease [17]. One may wonder why this nonsignificant longitudinal association was found in the study by Fox et al. when a positive cross-sectional association is observed in the present study. Several possibilities may be considered, such as: AC exposure did not contribute to or was not a cause of the reduction in cognitive performance over time after the onset of AD; other predictors associated with cognitive impairment contributed more in the model applied in this study; the sample size was not sufficiently large to highlight a significant association; or the assessed relationship between ACB and the cognitive performance did not have a linear relationship. The relationship between exposure and health outcome may be more dependent on the way the AC exposure is assessed, and it remains difficult to quantify a real drug effect in the absence of a gold standard.

The current study has some limitations. The relationships between AC exposure and outcomes are cross-sectional and do not allow the determination of a causal relationship. It is therefore difficult to determine whether the AC exposure may represent a higher risk for adverse health conditions; however, previous studies and recommendations tend to show that there is a relationship in this sense. The data collection contained all medications at inclusion without any distinction between what has been prescribed before and what has been prescribed at the memory clinic. The relationships were not adjusted for all comorbidities other than the main neurological diagnosis in the memory clinic and hypertension, diabetes mellitus, and hypercholesterolemia due to underreporting in the memory clinic; nevertheless adjustment was performed for the total number of drugs which may reflect the overall health condition and comorbidities managed by pharmaceutical therapeutics. This study included multiple testing which may increase the risk of concluding erroneously that there is a difference. Controlling for multiple testing is still debated as it leads to increasing the risk of not showing a difference that exists; we have chosen to report the initial results [45]. The missing data have not been replaced which can lead to a bias in the results in case the bias is dependent on the characteristics of the patients. Nevertheless, we believe that the presence of missing data in this study is mainly due to the constraints during the consultation, such as the lack of time for the staff to evaluate the scales, and may not linked with the characteristics of the patients. This probably did not influence the relevance of the results. The results of this study should be interpreted taking into account a possible selection bias of the study population since it is monocentric, consisting of all patients visiting a memory clinic at a given time and with at least one drug prescribed. However, medical care and assessment of patients is carried out in a similar way as in other memory clinics in France. Indeed, the center participates in the standardized collection of the French National Alzheimer database in routine care, which has been set up following the recommendations of the 2008–2012 French Alzheimer plan [46]. Patients without prescribed drugs were not included in this study, which may lead to a biased prevalence of patients with AC drugs [47]. Nonselected patients may then either have a better level of health or, to the contrary, at be a lower level if they have comorbidities that are not being treated by pharmaceutical therapy. The observed results of the present study may be generalized to other populations presenting with similar characteristics such as outpatients of memory clinics with cognitive complaints, at all stages of diagnosis, and with at least one prescribed drug. The AC scales are based on expert advice and partially upon the in vitro SAA. Therefore, the SAA limitations overlap those of drug scales. Moreover, AC scales have some additional drawbacks partly caused by the summation of each anticholinergic potential of each drug and the failure to take into account the dosage. It is unknown whether the cumulative exposure to AC drugs may be explained by linear additive models. Anticholinergic effects are dose-dependent and may not be proportional. Besides, the models do not take into account the specificity of the drugs for different muscarinic receptor subtypes. They also do not consider the possible synergistic or tolerance effects of different drugs. In addition, the comparison between scales is difficult because both the number and rating of the drugs listed varies considerably. The duration of medication use was not recorded. Another drawback to all the anticholinergic scales is the lack of taking into account the variability of the drug distribution to the brain. Anticholinergic risk assessment among the elderly is influenced by numerous features of the geriatric population such as neurodegenerative diseases, polypharmacy, and the different ageing process, which increases the diversity of drug distribution in the brain [48]. Finally, regarding the development of the ARS scale, it does not take into consideration the impact of some routes of administration such as topical, ophthalmic, otology, and inhaled medication preparations that may impact the AC exposure.

## Conclusion

In a population of patients with SCD or NCD, AC exposure is associated with functional impairment. The association between AC exposure and cognitive performance or behavioral disturbance varies according to the means of measurement of AC burden. This study highlights the difficulty to choose a good AC tool to accurately measure the AC exposure in order to prevent the risk of adverse events linked with AC exposure. Nevertheless, particular attention should be paid when prescribing drugs with AC properties, especially among patients with memory complaints, and during any stage of the disease. The identification of drugs with anticholinergic effects, which are not used for this purpose, should be performed in usual practice in order to improve the pharmaceutical management of patients with memory disorders. Optimization of drug prescription, including therapeutic alternatives, could be helpful for physicians. Interventional studies may be conducted to assess the effect of reduction of AC drug prescription on health outcomes.

## Abbreviations

AC: Anticholinergic
ACB: Anticholinergic Cognitive Burden
ADRD: Alzheimer’s disease or related diseases
ADS: Anticholinergic Drug Scale
ARS: Anticholinergic Risk Scale
BPSD: Behavioral and psychological symptoms of dementia
IADL: Instrumental Activities of Daily Living
MCI: Mild cognitive impairment
MMSE: Mini Mental State Examination
NCD: Neurocognitive disorders
NPI: Neuropsychiatric Inventory
SAA: Serum anticholinergic activity
SCD: Subjective cognitive decline
